# The role of ultrasound in the nucleation kinetics and Modification of product properties of 3-Nitro-1,2,4-triazol-5-one

**DOI:** 10.1016/j.ultsonch.2026.107744

**Published:** 2026-01-18

**Authors:** Xingquan Hu, Hao Wu, Pei Chang, Yiying Zhang, Cheng Xu, Lianjie Zhai, Bozhou Wang

**Affiliations:** State Key Laboratory of Fluorine and Nitrogen Chemicals, Xi’an Modern Chemistry Research Institute, Xi’an 710065, China

**Keywords:** Ultrasonic irradiation, NTO explosive, Nucleation kinetics, Metastable zone width, Solid–liquid interfacial tension

## Abstract

3-Nitro-1,2,4-triazol-5-one (NTO), a well-known energetic material, is extensively employed in the field of insensitive munitions. However, its irregular crystal morphology and broad particle-size distribution hinder its wider application. Ultrasonic-assisted crystallization offers an innovative approach to enhance the overall particle performance of NTO. In this study, NTO was subjected to ultrasound-assisted cooling crystallization using water as the solvent to control the crystal size and morphology, as well as remove adhered nitric acid and ensure environmentally production. The metastable zone width (MSZW) of NTO in aqueous solution was subsequently measured to understand the nucleation kinetics, revealing a significant reduction under ultrasonic irradiation. Employing Sangwal’s three-dimensional nucleation theory, the nucleation kinetic parameters were calculated. The results indicate that ultrasound affects the MSZW through reduction of the solid–liquid interfacial tension, promotion of burst nucleation, and suppression of particle agglomeration. Characterization of the ultrasound-processed NTO demonstrated a more regular morphology, disrupted agglomerates, reduced particle size, and a narrower particle-size distribution without altering the crystal polymorph. Compared with NTO raw material, the material demonstrates enhanced flowability and a 60% reduction in impact sensitivity.

## Graphical abstract

**Figure:**
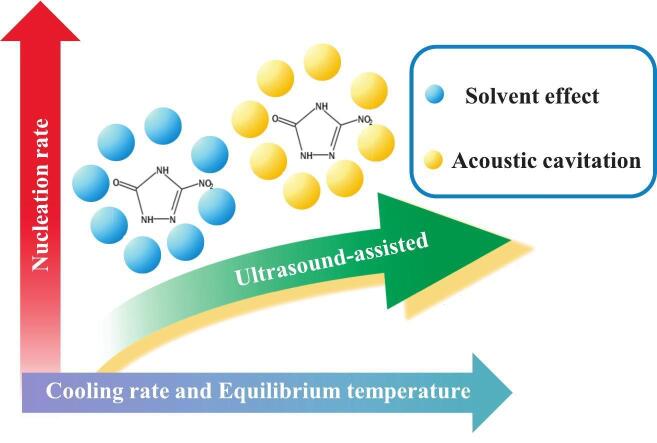

## 1 Introduction

Globally, the safety ammunition technology centered around insensitive energetic materials 3-nitro-1,2,4-triazol-5-one (NTO), as the most renowned representative among such materials, is favored by researchers due to its low sensitivity and high energy detonation velocity, pressure, and detonation energy that are comparable to those of cyclotrimethylenetrinitramine (RDX). These properties have facilitated its extensive application in the NTO-based insensitive explosive formulations, thereby advancing the insensitivity of safety ammunition [1]. Furthermore, the synthesis process of NTO is straightforward and cost-effective, making it suitable for large-scale industrial production. Consequently, NTO has significant application potential and has demonstrated broad prospects in cast PBX explosives, melt-cast explosives, and pressed explosive formulations [2].

However, the irregular crystal morphology and crystal agglomeration of pristine NTO materials remain critical issues urgently requiring resolution, resulting in low flowability and heightened sensitivity to sudden shock, which restrict their application in munitions filling processes [3], [4], [5]. Existing studies have shown that NTO crystallization in aqueous solution tends to form rod-like crystal aggregates. In the aqueous mixed solvent system, the formation of spherical aggregates can be observed [2], [6], [7], [8]. For example, Miao et al.’s study on toluene as a bridging agent in the water–N-methylpyrrolidone (NMP) system shows that the water component plays a key role in promoting the formation of spherical aggregates [2]. At the same time, introducing ultrasound suppresses the formation of dendrites during NTO crystallization, and together with the coupling effect of toluene the formation of smooth spherical aggregates. In addition, the solubility of NTO in different pure solvents (e.g., deionized water, methanol, ethanol, isopropyl alcohol, n-butanol, formic acid, ethyl acetate, acetonitrile, toluene, n-hexane) and binary solvent systems (e.g., ethanol + water, nitric acid + water, NMP + water) has also been systematically determined, and the data show that its solubility in water increases significantly with the increase of temperature in the range of 278.15 K to 328.15 K. Inspired by these researches, we can found that water can be selected as a good solvent for NTO crystallization processes [9], [10]. Meanwhile, further exploration is still required to modify NTO crystals with desired morphology and prevent crystal agglomeration.

In solution crystallization, conventional strategies to suppress crystal agglomeration and thereby control morphology and particle size include switching solvent systems, increasing agitation rates, adding surfactants. These existing approaches, however, have notable limitations: excessive agitation frequently induces crystal breakage, and the surfactant screening process is time-consuming with effects that are difficult to predict. Especially in terms of inhibiting agglomeration, ultrasonic radiation shows unique potential as a synergistic or alternative to the above traditional strategies. By contrast, ultrasonic irradiation offers multiple advantages — it can markedly accelerate nucleation [11], [12], [13], effectively inhibit agglomeration [12], narrow the crystal size distribution (CSD) [14], [15], and concomitantly improve product purity and morphological uniformity [16], [17]. Notably, in water-based NTO crystallization systems, reliance solely on conventional agitation control and cooling-program optimization is insufficient to stably produce crystals with regular morphology and uniform particle size [1], whereas the introduction of ultrasonic techniques can effectively overcome this process bottleneck [18], [19]. At present, the principal constraint on the deeper application of this technology is the lack of thorough fundamental research on ultrasound-assisted NTO crystallization: key crystallization kinetic parameters (such as nucleation and growth rates) remain to be quantified, and the mechanisms by which the ultrasonic field governs morphological evolution of crystals have not yet been clarified [20].

Nucleation, as the critical initiating step of the crystallization process, profoundly influences key aspects such as chiral separation [21], [22], [23], polymorph selection [24], and particle size control [25], [26]. The width of the metastable zone of supersaturation (MSZW) is a core parameter for investigating nucleation behavior and optimizing crystallization processes, as it directly reflects the nucleation kinetics of the system [27], [28], [29]. To elucidate crystallization mechanisms, researchers have endeavored to construct mathematical models that quantitatively relate the MSZW to cooling rate or saturation temperature [30], [31]. The studies by Sangwal et al. [32], [33] combined classical nucleation theory with solution theory and proposed a linear relationship between the maximum undercooling and the logarithm of the cooling rate; this conclusion has been validated by MSZW data for NTO in water obtained using the KIM [29] polythermal method, significantly enhancing the model’s physical interpretability. In recent years, this theory has received further experimental support in the crystallization systems of various compounds [34], [35] and under ultrasonic field conditions [36].

This study initiates a systematic investigation into the continuous ultrasound-enhanced nucleation process of NTO. The MSZW under varying ultrasound power densities is quantitatively analyzed, followed by the estimation of nucleation kinetic parameters derived from MSZW datasets through the application of Sangwal's three-dimensional nucleation theory. Subsequently, employing water as an environmentally benign solvent, controlled crystallization of NTO is achieved with precise particle size modulation. Comprehensive characterization of ultrasonically assisted NTO crystals is further conducted, encompassing critical attributes including morphological profiles, thermal behavior, dispersion stability, bulk density, and mechanical sensitivity. These findings provide a potential approach for modulating the performance of energetic materials and offer critical guidance for advancing their application in propellant and explosive formulations.

## 2 Experimental section

### 2.1 Materials

NTO raw material (purity > 99 %) is a white or light yellow solid powder, provided by Xi'an Institute of Modern Chemistry. Dseionized water, laboratory-made, conductivity ≤ 5.0 μS·cm^−1^.

### 2.2 MSZW measurements

Saturated NTO solutions at different temperatures (308.15, 313.15, 318.15, 323.15 and 328.15 K) were prepared based on reported NTO solubility data in water [10]. The solutions were stirred for 60 min at 5 K above the saturation temperature with a magnetic stirrer (HS7, IKA mixing, and then filtered through a 0.45 μm membrane. A 150 mL aliquot of the filtrate was transferred to a 250 mL jacketed crystallizer and held for 10 min at the saturation temperature. Temperature was controlled by a heating–cooling circulator (CF41, Julabo, Germany), and the solution temperature in the crystallizer was measured with a thermocouple having an accuracy of 0.1 K. Turbidity was monitored with a turbidity probe (InPro 8200, Mettler–Toledo, Switzerland). Under constant cooling rates of 6, 30, 60 and 90 K·h^−1^, with the magnetic stirring rate maintained at 500 rpm was considered to have occurred when the turbidity began to increase continuously; the corresponding temperature was recorded as the nucle temperature (see Supporting Information 1.3). The difference between the saturation temperature (T_0_) and the nucleation temperature (T_1_) is MSZW (ΔT_max_). To reduce experimental variability and confirm statistical reliability, all processes were repeated three times independently [37].

**Fig. 1:**
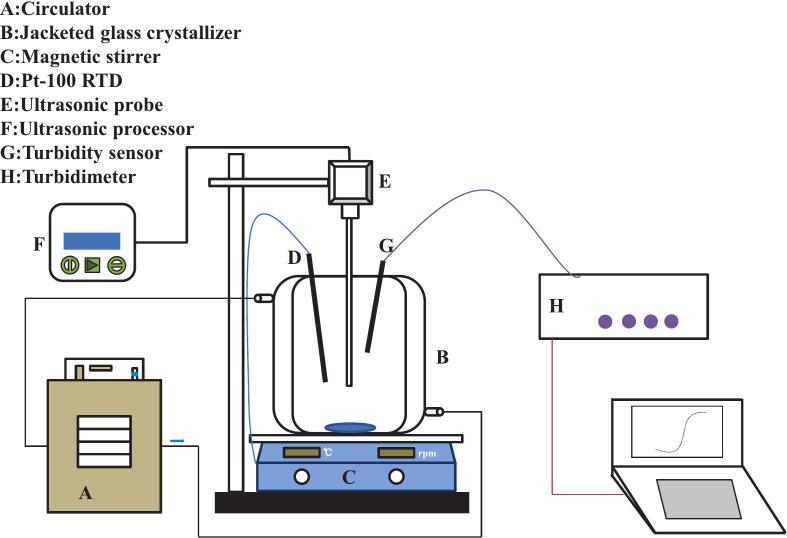
Schematic diagram of experimental setup.

The ultrasound irradiation frequency was set to 20 kHz. The ultrasonic processor used was a GM4200 (Julabo, Germany), equipped with a 6 mm diameter probe and a calculated effective radiating area of 1.131 cm^2^, and was employed to perform continuous irradiation (100 % duty cycle) at varying cooling rates, ultrasonic powers (20 ∼ 80 W), and saturation temperatures to determine the metastable zone width (MSZW). The volume of NTO solution in each crystallization experiment was 150 mL. A schematic diagram of the experimental apparatus is shown in Fig. 1.

### 2.3 Solid characterization

Powder X-ray diffraction (PXRD) analysis was conducted on both the raw material and samples from the solid–liquid-phase equilibrium systems under various conditions. The PXRD measurements utilized Cu-Kα radiation (0.15405 nm) within a diffraction angle (2*θ*) range of 5.0 to 40.0°, with a scanning rate of 5°·min^−1^.

To examine the morphology of NTO, a Hitachi S-3400 N scanning electron microscope and a Nikon ECLIPSE Ti2 optical microscope were employed.

Thermal properties of NTO were assessed using a Netzsch DSC-204 differential scanning calorimeter, with measurements performed on a 0.5 mg sample heated from 323.15 K to 673.15 K at a ramp rate of 600 K·h^−1^. The mechanical sensitivity of NTO was evaluated using the explosion-probability method.

## 3 Model of MSZW

According to the regular solution theory, the relationship between *c*_0_ and *c*_1_ can be expressed by (1), where *c*_0_ and *c*_1_ denote the solution concentrations at the saturation temperature *T*_0_ and the nucleation temperature *T_1_*.

(1) $$\text{ln}\frac{c_{0}}{{\text{c}}_{\text{1}}}=\frac{\Delta {\text{H}}_{\text{s}}}{{\text{R}}_{\text{g}}{\text{T}}_{\text{1}}}\frac{\Delta {\text{T}}_{\text{max}}}{{\text{T}}_{\text{0}}}\;$$

When the rate of change of supersaturation (*σ*) satisfies *σ* ≪ 1, the equation ln*S* ≈ *σ* = Δ*c*/*c*_1_ holds, where *S* = *c*_0_/*c*_1_. Rearranging this equation gives:

(2) $$\text{ln}\text{S}=\frac{\Delta c}{c_{1}}=\frac{\Delta {\text{H}}_{\text{s}}}{{\text{R}}_{\text{g}}{\text{T}}_{\text{1}}}\frac{\Delta {\text{T}}_{\text{max}}}{{\text{T}}_{\text{0}}}\;$$

Sangwal further postulates an association between the nucleation rate, denoted as J, and the relative change in solution supersaturation (Δ*c*/*c*_1_) [33]. Within this theoretical framework, the parameter Δ*H*_s_ is defined as the compound’s enthalpy of dissolution. Under the experimental conditions of this study (308.15 K ∼ 328.15 K), the enthalpy of dissolution of NTO in water was calculated to be 26.864 × 10^3^ J·mol^−1^; the detailed calculation procedure is provided in Section 1.2 of the Supporting Information. Consequently, the nucleation rate expression can be formulated in the following manner:

(3) $$\text{J}=\text{f}\frac{\Delta \text{c}}{{\text{c}}_{\text{1}}\Delta \text{t}}=\text{f}\frac{\Delta \text{c}}{{\text{c}}_{\text{1}}\Delta \text{T}}\frac{\Delta \text{T}}{\Delta \text{t}}=\text{f}\frac{\Delta {\text{H}}_{\text{s}}}{{\text{R}}_{\text{g}}{\text{T}}_{\text{1}}}\frac{\text{R}}{{\text{T}}_{\text{0}}}\;$$

The cooling rate, denoted as *R*, and the proportionality constant *f* represent the volumetric density of entities (e.g., particles, ions, or clusters), whose dynamics are governed by aggregation and diffusion mechanisms within the solution. By integrating Equation (3) with classical three-dimensional nucleation theory, while designating T_1_ as the nucleation temperature and substituting *T_0_* for *T_2_*, the formation rate of stable three-dimensional spherical nuclei can be derived as follows:

(4) $$\text{Aexp}\left[\left(\frac{\text{-16[GSLPI]}\gamma^{\text{3}}{\text{V}}_{\text{s}}^{2}}{\text{3}{\text{k}}^{\text{3}}{\text{T}}_{\text{1}}^{\text{3}}}\right){\left(\frac{{\text{R}}_{\text{g}}{\text{T}}_{\text{1}}}{\Delta {\text{H}}_{\text{s}}}\right)}^{2}{\left(\frac{{\text{T}}_{\text{0}}}{{\Delta \text{T}}_{\text{max}}}\right)}^{2}\right]=\text{f}\frac{\Delta {\text{H}}_{\text{s}}}{{\text{R}}_{\text{g}}{\text{T}}_{\text{1}}}\frac{\text{R}}{{\text{T}}_{\text{0}}}\;$$

Where *k* denotes the boltzmann constant with value 1.381 × 10^−23^ J·K^−1^; *R_g_* denotes the gas constant with value 8.314 J·mol^−1^·K^−1^; and *V_s_* denotes the molecular volume with value 1.121 × 10^−28^ m^3^.

When taking logarithm on both sides of eq (3) and further simplifying, equation (4) can also be rewritten as equation (5)

(5) $${\left(\frac{{\text{T}}_{\text{0}}}{{\Delta \text{T}}_{\text{max}}}\right)}^{2}=\left[\frac{\text{3}{\text{k}}^{\text{3}}{\text{T}}_{\text{1}}^{\text{3}}}{\text{16[GSLPI]}\gamma^{\text{3}}{\text{V}}_{\text{s}}^{2}}{\left(\frac{\Delta {\text{H}}_{\text{s}}}{{\text{R}}_{\text{g}}{\text{T}}_{\text{1}}}\right)}^{2}\right]\left(\text{ln}\frac{\text{A}{\text{R}}_{\text{g}}{\text{T}}_{\text{1}}}{\text{f}\Delta {\text{H}}_{\text{s}}}+\text{ln}{\text{T}}_{\text{0}}\text{-}\text{ln}\text{R}\right)\;$$

The equation is further simplified to highlight the relationship between (*T*_0_/Δ*T*_max_)^2^ and ln *R*, and equation (6) is obtained.

(6) $${\left(\frac{{\text{T}}_{\text{0}}}{\Delta {\text{T}}_{\text{max}}}\right)}^{2}={\text{F}}_{\text{1}}\left(\text{X}+\text{ln}{\text{T}}_{\text{0}}\text{-}\text{ln}\text{R}\right)=\text{F}\text{-}{\text{F}}_{\text{1}}\text{ln}\text{R}\;$$

With the intercept of *F* = *F*_1_(*X* + ln*T*_0_) and slope of *F*_1_, where

(7) $${\text{F}}_{\text{1}}=\left[\frac{\text{3}{\text{k}}^{\text{3}}{\text{T}}_{\text{1}}^{\text{3}}}{\text{16[GSLPI]}\gamma^{\text{3}}{\text{V}}_{s}^{2}}{\left(\frac{\Delta {\text{H}}_{\text{s}}}{{\text{R}}_{\text{g}}{\text{T}}_{\text{1}}}\right)}^{2}\right]\;\;$$

(8) $$X=\text{ln}\left(\frac{\text{A}{\text{R}}_{\text{g}}{\text{T}}_{\text{1}}}{\text{f}\Delta {\text{H}}_{\text{s}}}\right)\;$$

Equation (6) reflects a linear relationship between (*T*_0_/Δ*T*_max_)^2^ and ln *R* at a given initial saturation concentration. The slope *F*_1_ is influenced by the combined effects of the thermodynamic parameters *γ*, *T*_1_, and Δ*H*_s_. The true driving force of the crystallization process is the difference in chemical potential (*μ*_1_) in the liquid phase and the chemical potential (*μ_s_*) in the solid phase. Combining the classical nucleation theory with the ln*S* approximation hypothesis, Δμ can be calculated using Equation (9) [28].

(9) $$\Delta \mu =\mu_{1}-\mu_{s}\approx \Delta \mu^{\prime }=\text{kT}\text{ln}\text{S}=\text{k}\frac{\Delta {\text{H}}_{s}}{{\text{R}}_{\text{g}}}\frac{\Delta {\text{T}}_{\text{max}}}{{\text{T}}_{\text{0}}}\;$$

Based on CNT, the critical nucleation size is $r_{\mathrm{crit}}$

(10) $${\text{r}}_{\text{crit}}=\frac{\text{2}\gamma {\text{V}}_{s}}{\Delta \mu }=\frac{\text{2}\gamma {\text{R}}_{\text{g}}{\text{V}}_{s}}{\text{k}\Delta {\text{H}}_{s}}\frac{{\text{T}}_{\text{0}}}{\Delta {\text{T}}_{\text{max}}}\;$$

Analyzing equations (9), (10), we can find the following relationship between $r_{\mathrm{crit}}$ and Δ*μ*:

(11) $$\text{ln}{\text{r}}_{\text{crit}}=-\text{ln}\Delta \mu \text{+ln(2}\gamma {\text{V}}_{s}\text{)}\;$$

## 4 Results and discussion

### 4.1 MSZW of NTO without ultrasound.

**Fig. 2:**
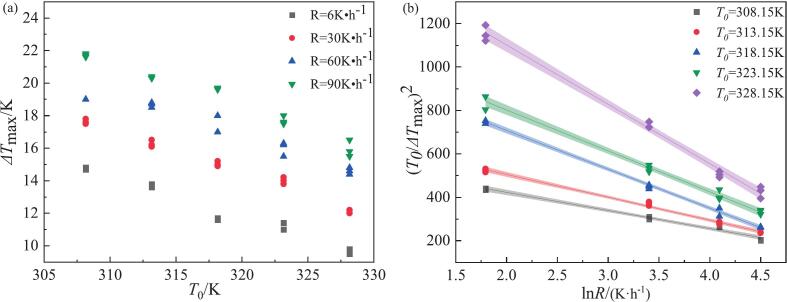
(a) NTO in water at different cooling rates as a function of saturation temperatures in the MSZW; (b) Relationship between MSZW and the cooling rate at different saturation temperatures in water according to Sangwal’s theory.

In the water crystallization of NTO, the effects of cooling rate (*R*) and saturation temperature (*T*_0_) on the MSZW were systematically examined; the experimental results are summarized in Fig. 2(a) and Table S1. At a fixed saturation temperature, the MSZW increased markedly with increasing cooling rate, a behavior consistent with that reported for many organic solutes [28], [35]. Conversely, when the cooling rate was held constant, the MSZW decreased as the saturation temperature increased. Within the experimental range studied, the MSZW broadening induced by higher cooling rates was more pronounced than the narrowing produced by elevated saturation temperatures.

These trends can be rationalized by considering the interplay between supersaturation generation and nucleation kinetics. A higher saturation temperature corresponds to a higher solute concentration in solution, thereby increasing the frequency of solute–solute collisions, which may promote nucleation and consequently reduce the MSZW [38]. Conversely, increasing the cooling rate lowers the nucleation temperature and drives a more rapid accumulation of supersaturation, thereby broadening the MSZW. At sufficiently high cooling rates, the rate of supersaturation generation may exceed the rate of cluster growth; under such conditions, the formation of stable crystal nuclei may require a longer relaxation time.

To quantify this behavior in the absence of ultrasound, Sangwal’s theory was applied and the results are presented in Fig. 2(b). A clear linear relationship was obtained between (*T*_0_/Δ*T*_max_)2 and ln R across the examined temperature range. The slopes of these linear fits increased with saturation temperature, indicating that the cooling rate exerts a progressively stronger influence on the MSZW at higher *T*_0_. Moreover, the fitted lines for different *T*_0_ values tend to converge toward a common intersection. The convergence of the fitted lines implies a regime in which the MSZW becomes predominantly governed by the cooling rate rather than by the saturation temperature. In practical terms, beyond a certain cooling-rate threshold, the effect of *T*_0_ on the MSZW of NTO is negligible.

### 4.2 MSZW of NTO with ultrasound.

#### 4.2.1 Effect of ultrasound on the MSZW

**Fig. 3:**
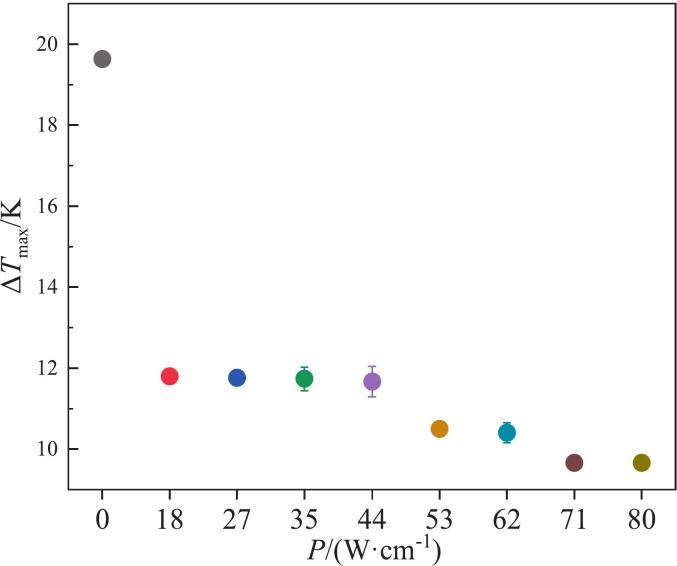
Effect of ultrasonic power densities on the MSZW at an initial temperature of 318.15 K and a cooling rate of 90 K·h^−1^.

Without ultrasound, the MSZW of NTO is exceptionally broad, which hinders the triggering and control of nucleation events regardless of variations in the crystallization system’s intrinsic parameters [39], [40], [41], [42]. Inspired by studies examining the influence of ultrasound on both MSZW and nucleation, we measured the MSZW at several saturation temperatures and present the results in Table S1 ∼ S2. To establish reasonable ultrasonic conditions, the following steps were taken: first, the effects of ultrasonic power densities in the range of 18 W·cm^−1^ ∼ 80 W·cm^−1^ were preliminarily screened. Table S2 lists the mesostability region under various conditions and shows that ultrasonic powers at 308.15 K and 313.15 K have little effect on MSZW (no significant difference). Consequently, data at 318.15 K, where significant differences were observed, were plotted in Fig. 3, and the average ΔT_max_ for the 18 W·cm^−1^ condition is reported in Table 1.

**Table 1:** The MSZW of NTO with ultrasonic irradiation 18 W·cm^−1^.

	Δ*T*_max_ /K				
R	*T*_0_ = 308.15 K	*T*_0_ = 313.15 K	*T*_0_ = 318.15 K	*T*_0_ = 323.15 K	*T*_0_ = 328.15 K
6	11.7	10.8	8.3	7.4	7.3
30	13.6	12.9	9.8	8.9	8.8
60	14.6	14.2	10.8	9.7	9.6
90	16	14.9	11.8	10.9	10.5

Fig. 3 indicates that the introduction of ultrasound can significantly reduce the MSZW, and as the ultrasonic power density increases, the MSZW decreases monotonically. Combined with Table 1, it can be seen that under the same cooling rate and initial temperature, the MSZW of NTO under ultrasound is significantly lower than under no-ultrasound conditions, further confirming that ultrasound can promote nucleation. The mechanism is as follows: cavitation bubbles induced by ultrasound repeatedly grow and collapse, creating transient highly supersaturated microenvironments and releasing energy that helps overcome the critical barrier for spontaneous nucleation; simultaneously, bubble interfaces provide additional attachment sites for heterogeneous nucleation, thereby enhancing nucleation [17]. In addition, the fluid shear resulting from bubble collapse strengthens fluid mixing, thins the diffusion boundary layer between the crystal surface and the bulk phase, increases molecular collisions and mass transfer rates, and further raises the nucleation rate [43]. Therefore, as the ultrasonic power density increases, cavitation intensifies [44], shear and microturbulence are enhanced, nucleation becomes easier, and the MSZW continues to decrease.

#### 4.2.2 Analysis of metastable zone width data for calculation of nucleation rate

**Fig. 4:**
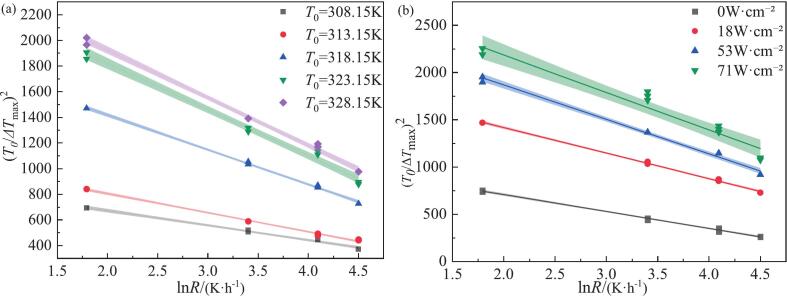
According to Sangwal's theory, the MSZW model of NTO under ultrasound irradiation: (a) different initial temperatures; (b) different ultrasound powers under the same initial temperature of 318.15 K cooling rate of 90 K·h^−1^.

In order to further elucidate the relationship among saturation temperature, cooling rate and MSZW under varying ultrasonic conditions, the Sangwal model was employed. As illustrated in Fig. 4, a perfect linear correlation is observed between (*T*_0_/Δ*T*_max_)^2^ and ln R. The introduction of ultrasound leads to a reduction in Δ*T*_max_, thereby increasing the value of (*T*_0_/Δ*T*_max_)^2^ compared with the non-ultrasonic case. Both slope and intercept are sensitive to *T_0_*, rising concomitantly with *T_0_*. Table 2 shows that under ultrasonic conditions the values of F and F_1_ are substantially larger than those in the absence of ultrasound. Furthermore, as ultrasonic power density increases, MSZW decreases and the values of F and F_1_ correspondingly increase. According to Equation (6), the slope and intercept are derived from the fitted line and utilized to calculate the solid–liquid interfacial tension at various cooling rates and saturation temperatures, respectively.

**Table 2:** Nucleation parameters calculated according to Sangwal’s theory.

condition	*T*_0_/K	Slope (−*F*_1_)	Intercept (*F*)	R^2^	*γ*/(mJ/m^2^)	*f*/A
Non-ultrasonic	308.15	−83.20 ± 3.13	588.69 ± 11.26	0.986	60.21	24.06
	313.15	−105.51 ± 2.85	716.46 ± 10.25	0.993	55.63	33.19
	318.15	−166.39 ± 3.51	1056.93 ± 12.65	0.98	47.79	53.39
	323.15	−188.34 ± 5.70	1178.71 ± 20.51	0.991	45.86	60.76
	328.15	−273.35 ± 7.99	1647.94 ± 28.75	0.992	40.50	79.32
Ultrasonic	308.15	−115.01 ± 3.45	902.82 ± 12.42	0.996	54.05	11.26
	313.15	−148.99 ± 2.54	1103.56 ± 9.15	0.999	49.58	18.16
	318.15	−270.62 ± 3.70	1959.18 ± 13.32	0.999	40.64	22.4
	323.15	−359.03 ± 10.75	2537.7 ± 38.67	0.996	36.98	27.52
	328.15	−371.77 ± 7.26	2668.05 ± 26.11	0.998	36.56	25.49

**Fig. 5:**
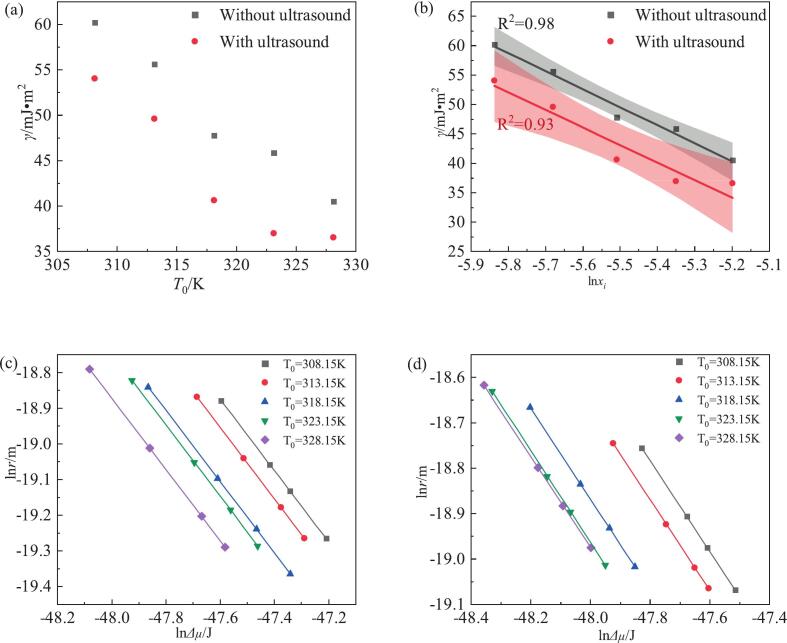
NTO nucleation parameters according to Sangwal's theory: (a) relationship between solid–liquid interfacial tension and initial temperature; (b) relationship between solubility and solid–liquid interfacial tension; (c) Relationship between thermodynamic driving forces (calculated) and critical core size (calculated) at different saturation temperatures in the absence of ultrasound; (d) The relationship between the thermodynamic driving force (calculated value) and the critical core size (calculated value) at different saturation temperatures under ultrasonic irradiation at 18 W·cm^−1^;

What are the intrinsic factors affecting nucleation? The relationship among the solid–liquid interfacial tension (γ), the driving force, and the saturation temperature was investigated; this relationship is shown in Fig. 5 and Table 2. The results indicate that the solid–liquid interface exerts a significant influence on nucleation behavior, implying that a portion of nucleation events may occur heterogeneously at or in the vicinity of this interface. In this case, the use of homogeneous classical nucleation theory (CNT) should be regarded as an effective, averaged description. Notably, γ decreases with increasing saturation temperature, and increasing solubility likewise reduces γ. The fundamental reason that the MSZW of NTO can be effectively narrowed is the enhanced capacity to form the solid–liquid interface. Higher solubility implies a greater number of solute molecules and an increased probability of collisions in a supersaturated solution; this can readily reduce the work required to generate the *γ* and increase the probability of binding between solute molecules, thereby facilitating nucleation Under ultrasonic conditions, the rapid collapse of cavitation bubbles induces concentration gradients (transport effects) in the vicinity of the implosion [45], increasing local supersaturation and lowering the γ value during nucleation. Simultaneously, cavitation bubbles can act as active nucleation centers that promote local nucleation. This conclusion aligns with the findings of Granberg [46] who assert that the solid–liquid interfacial tension of the acetaminophen-acetone–water system tends to rise with decreasing solubility. Furthermore, Bennema [47] has demonstrated a linear relationship between *γ* and solubility ln *x* in aqueous solutions. A comparison of sonication conditions revealed that the MSZW decreased with increasing solubility.

When considering the effects of ultrasound, it has been established that ultrasound can generate localized high pressures. Under these conditions, cavitation leads to transient increases in local supersaturation or promotes the formation of solid–liquid interfaces; this can be attributed to ultrasound-induced changes in the crystallization driving force, which in turn affect the system’s ability to generate solid–liquid surfaces. As shown in Table 2 and Fig. 5 (the relationship between *γ* and *T*_0_), *γ* decreases with increasing saturation temperature *T*_0_; under 18 W·cm^−1^ ultrasound, with initial concentration and cooling rate held constant, the *γ* is lower than in the absence of ultrasound, with an average reduction of 19.36 %. During the crystallization of NTO in the aqueous phase, the MSZW is relatively large; the introduction of ultrasound significantly increases the crystallization driving force Δ*μ* (Fig. 5c–d), thereby effectively enhancing the probability of intermolecular collisions, narrowing the MSZW, and promoting nucleation.

Within the framework of classical nucleation theory (CNT), calculations based on Equations (9), (10) indicate that both the critical nucleation radius and the critical nucleus size exhibit a linear relationship with the thermodynamic driving force; as the driving force increases, the critical nucleus radius decreases slightly. Although the Δ*μ* and *r*_crit_ obtained from Equations (9), (10) are approximate, they nonetheless provide valuable references for analyzing the influence of ultrasound on the nucleation process. Fig. 5 reveals that the nucleation size distributions of NTO under non-ultrasonic and ultrasonic conditions are almost identical: under non-ultrasonic conditions the nucleation sizes range from 4.2 nm to 6.9 nm (corresponding to ln(*r*) /m ≈ −19.3 to − 18.7), while under ultrasonic conditions they range from 5.7 nm to 8.2 nm (corresponding to ln(*r*) /m ≈ −19.1 to − 18.6). The fitted lines appear to be laterally shifted, indicating that during the nucleation of NTO in aqueous solution, ultrasound, cooling rate and initial temperature primarily affect the nucleation driving force and have only minor effects on the nucleation size.

### 4.3 Effects of ultrasound on the Crystalline products of NTO

#### 4.3.1 Influence of ultrasonic irradiation on the particle size and morphology of the products

Crystallization of NTO in water was carried out not only to remove nitric acid adhering from the synthesis but also to regulate the product's particle size and morphology. Comparative crystallization experiments of the NTO solution were performed under various conditions, namely without ultrasound and with ultrasonic power densities of 18 W·cm^−1^, 35 W·cm^−1^, and 53 W·cm^−1^, and at cooling rates of 60.0 K·h^−1^. The cooling procedure employed in these experiments was identical to that used for the determination of the MSZW.

**Fig. 6:**
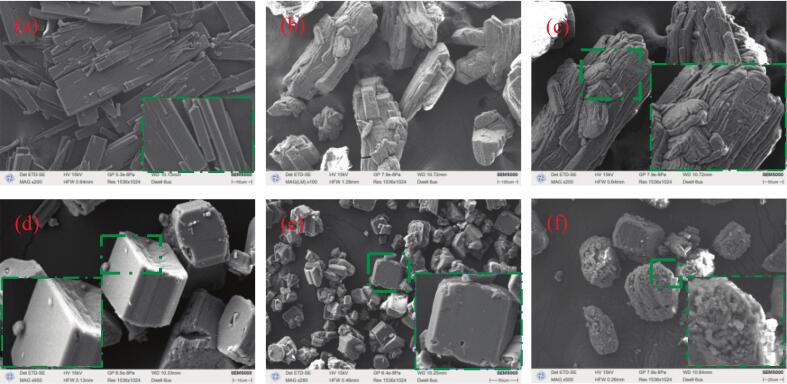
SEM images of product NTO under various process conditions: (a) Raw NTO; (b ∼ c) without ultrasound; (d) with ultrasound: 18 W·cm^−1^; (e) with ultrasound: 35 W·cm^−1^; (f) with ultrasound: 53 W·cm^−1^.

The morphological distribution of NTO crystals obtained under various crystallization conditions is primarily presented in Fig. 6. Without ultrasonic assistance, the morphology of NTO crystals mainly exhibits longitudinally elongated, rod-like agglomerations with frequent occurrences of spurs (Fig. 6.a). Such defect-laden NTO crystals are not ideal due to the abundance of spurs which increase sensitivity, making them prone to explosion from friction or impact while also adversely affecting downstream processing efficiency. In the absence of ultrasonic aid, NTO crystals primarily take on a rod-like shape in water, appearing as layered stacks and in some instances, asymmetrically piled up (Fig. 6.b-c). This phenomenon is mainly dictated by the solvent effect of water on the growth of the crystals, aligning with the data reported in literature [2], [48], [49]. However, with the application of ultrasonic irradiation, NTO crystals exhibit a distinctly angular, dispersed blocky morphology that effectively inhibits clustering. At lower ultrasonic power densities (18 W·cm^−1^, 35 W·cm^−1^), the formation of smaller crystals within the crystallized mass has not been observed. As the power density increases to 53 W·cm^−1^, due to the ultrasonic effect, the edges of the crystals begin to fragment, resulting in shattered crystals that are easily absorbed by larger ones.

**Fig. 7:**
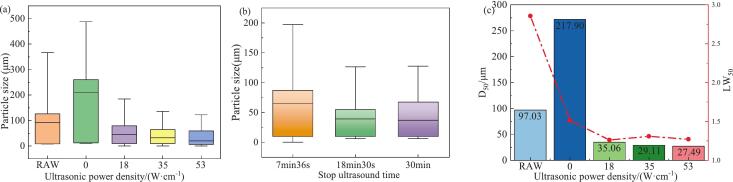
Effect of Ultrasonication Duration on Particle Size Distribution:(a) Effect of varying ultrasonic power density on particle size distribution;(b) Effect of sonication time on particle size distribution;(c) Effect of varying ultrasonic power density on the D_50_ and aspect ratio (LW_50_) of NTO.

According to particle size distribution analysis shown in Fig. 7, ultrasonic waves significantly reduce the particle size of NTO and lead to a more concentrated distribution. When NTO crystallizes in the aqueous phase, tend to coalesce or form outgrowths at crystal corners, resulting predominantly-rod aggregates with relatively large particles and a broad particle size distribution. Conversely, upon the introduction of ultrasound, cavitational effects increase the number of nucleation sites and promote extensive nucleation, thereby consuming the overall supersaturation of the solution. Miyasaka et al, proposed the possibility of controlling the crystal size of the final product by controlling the number of primary nucleation sites with appropriate ultrasonic energy [50]. Moreover, mechanical effects induce deagglomeration of NTO aggregates, fragmenting large crystals into smaller ones. We show in Fig. 7(b) the effect of ultrasonic time on particle size distribution at a cooling rate of 90 K·h^−1^ and an ultrasonic power density of 18 W·cm^−1^ during crystallization from 318.15 K to 273.15 K. Under shorter ultrasound durations, due to less consumption of supersaturation, crystal growth exceeds nucleation rate after stopping ultrasound, causing early nucleated crystals to grow larger, resulting in a broader particle size distribution; while the prolongation of ultrasound duration, even throughout the entire crystallization process, significantly narrows the particle size distribution, because continuous ultrasonic application drastically reduces the supersaturation, greatly enhancing nucleation rate far beyond the crystal's own growth rate.

#### 4.3.2 Evolution process and formation mechanism

**Fig. 8:**
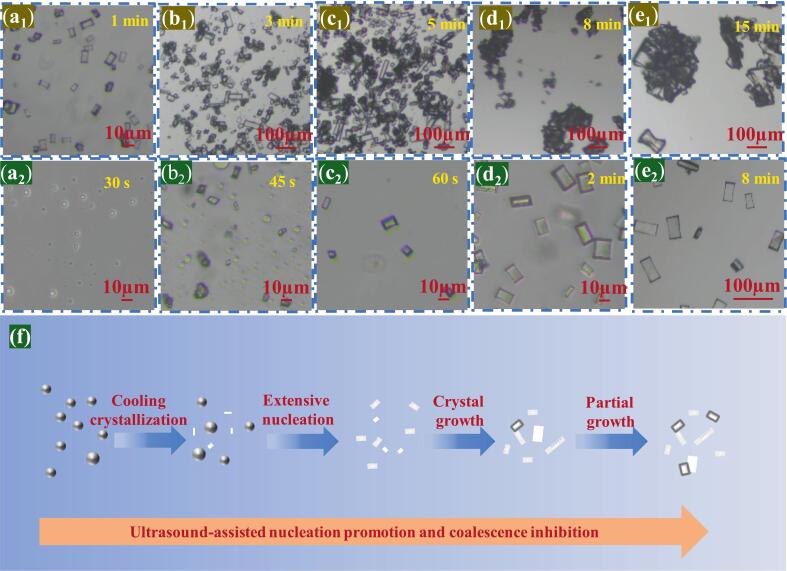
(a_1_–e_1_) Inverted optical micrographs capturing the aggregation process of NTO crystals. (a_2_–e_2_) Inverted optical micrographs capturing the ultrasound-assisted crystallization process of NTO. (f) Schematic representation of the NTO crystallization process under ultrasound, providing a visual model for each procedural step.

To further investigate the nucleation and agglomeration dynamics of NTO in aqueous media and the mechanism of morphology regularization under ultrasonic treatment, an inverted optical microscope were employed for offline sampling to monitor the crystallization of NTO at different time intervals. Based on prior determinations of the metastable zone, a gradient cooling program of 1 K·h^−1^ was applied. When samples were taken at the onset of nucleation, several translucent small crystals were observed (Fig. 8a1); these crystals were predominantly well-defined rectangular block-like prisms. As the temperature decreased, some nucleation events became concentrated at the crystal corners, leading to adhesion and the formation of aggregates (Fig. 8b1), with the edges losing translucency. Extensive nucleation at the corner regions further depleted the solution supersaturation, thereby retarding crystal growth so that only a few crystals developed into elongated rod-like morphologies (Fig. 8c1). However, with prolonged crystallization time, agglomeration intensified (Fig. 8d1 ∼ e_1_).

Under ultrasonic irradiation, numerous cavitation bubbles—colorless and transparent—form in the solution; their continual expansion and collapse can be observed by polarized light microscopy (Fig. 8a2 ∼ b_2_). Cavitation markedly enhances mass transfer and lowers the nucleation barrier, ultimately inducing extensive precipitation of NTO crystals. The majority of the crystals are optically clear and transparent (Fig. 8c2). The massive precipitation consumes a large portion of the supersaturation, thereby retarding crystal growth; only a small number of secondary-nucleated crystals adhere to the surfaces of the larger crystals, resulting in partial opacity at some crystal edges (Fig. 8d2–e2). Fig. 8(f) schematically depicts this ultrasonic crystallization process; in conjunction with the foregoing analysis of ultrasound-assisted NTO nucleation kinetics, these observations indicate that ultrasound promotes burst nucleation, with acoustic cavitation facilitating molecular desolvation and suppressing solvent-mediated crystal agglomeration. Overall, the crystallization of NTO under ultrasound is characterized by nucleation efficiency exceeding crystal growth, fostering the formation of NTO products with narrow particle size distributions and reduced particle size.

#### 4.3.3 Powder properties

**Fig. 9:**
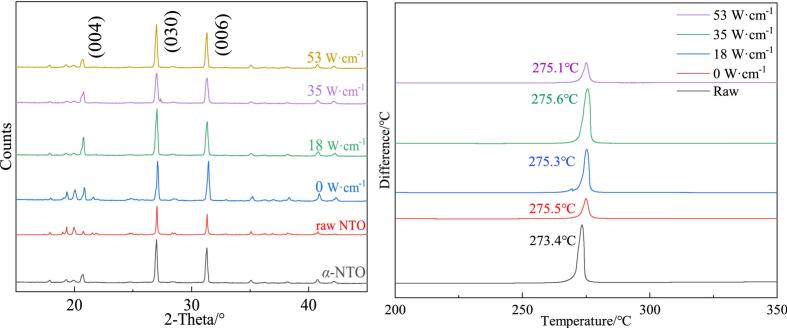
Crystal form and thermal properties of NTO crystals. (a) XRD patterns of NTO. (b) DSC curve.

NTO exists in three polymorphic forms: α, β, and γ, with α polymorph being the most commonly utilized, characterized by a triclinic crystal system (space group P1̅). The α-NTO crystal exhibits a density of 1.927 g·cm^−3^, a detonation velocity of 8700 m·s^−1^, and a detonation pressure of 34.9 GPa [2]. As shown in Fig. 9a, by comparing the PXRD patterns of the raw material and crystallized samples, it was observed that the diffraction peaks of raw NTO, α-NTO, and Ultrasonic-NTO appear consistently at 20.79°, 27.06°, and 31.41°, corresponding to the crystallographic planes (0 0 4), (0 3 0), and (0 0 6), respectively. The complete alignment of peak positions indicates that no new phase formation occurred during crystallization in aqueous media.

In the study of energetic materials, the measurement and analysis of thermal properties often employ differential scanning calorimetry (DSC). Research indicates that improvements in crystallinity and the integrity of the crystal structure directly affect thermal stability, while crystal defects (such as vacancies and dislocations) lead to a decrease in decomposition temperature, thereby reducing thermal stability [51], [52]. Data from Fig. 9 show that NTO exhibits only one decomposition peak temperature, and the decomposition peak temperature of recrystallized NTO is significantly higher than that of the raw NTO. This phenomenon indicates that crystallizing NTO in water (removing nitric acid), regardless of whether ultrasonic irradiation is used, can effectively enhance the crystallinity of its crystals and reduce defects within the crystals. This process not only improves the thermal stability of NTO but also provides a more reliable foundation for its safety and performance enhancements.

**Fig. 10:**
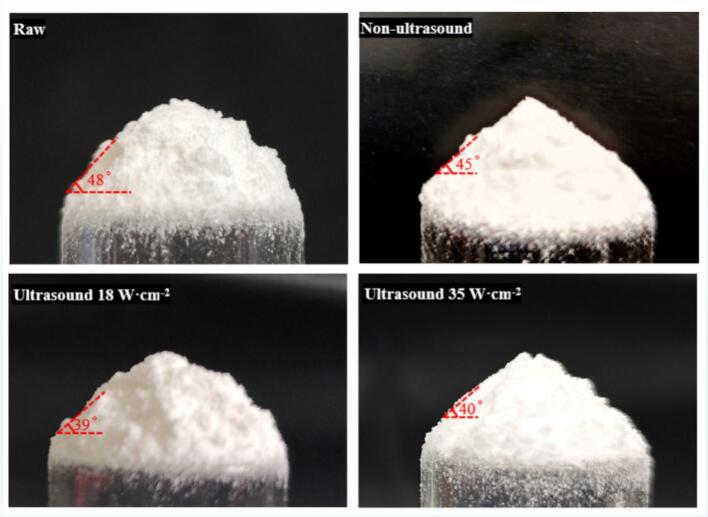
Measurement of the angle of repose for samples.

In transportation, casting, and filling operations, the flowability of explosive materials exerts a significant influence on their handling and application. Measurement of the angle of repose is a commonly employed method for assessing material flowability and is applicable to various flow regimes. By measuring an explosive’s angle of repose, its flowability can be effectively assessed: a smaller angle of repose corresponds to better flowability. In the present experiments, the flowability before and after crystallization was compared; along with the aforementioned comparison of crystal habits produced by different processing routes, the results indicate that the flowability of NTO changes upon crystallization, and these changes are shown in Fig. 10. As shown, the angle of repose of NTO decreases after crystallization, indicating improved flowability, with the NTO product subjected to ultrasonic treatment exhibiting a particularly marked enhancement. On one hand, explosive materials with a narrower particle size distribution exhibit reduced effective interparticle contact area, thereby decreasing interparticle friction and overall flow resistance and thus enhancing flowability. On the other hand, ultrasonic treatment suppresses agglomeration during crystallization, promoting dispersion of NTO particles and further improving flowability.

It is well-established within the energetic materials community that NTO exhibits exceptionally low sensitivity characteristics. Previous studies using the BAM method have quantitatively assessed this insensitivity, with reported impact sensitivity values of more than 50 J and friction sensitivity measurements of more than 360 N [53]. Both exceeded the upper limit of the standard test equipment, thus strongly confirming that NTO was classified as an insensitive explosive. In the present investigation, we conducted quantitative sensitivity assessments following Method 601.1 (Explosion Probability Method) of the Chinese National Military Standard GJB772A-97. The impact sensitivity was determined using a standard 10 kg drop hammer system, with drop heights corresponding to the characteristic height for a 50 mg sample. Friction sensitivity evaluations were performed on a WM-1 friction sensitivity tester under standardized conditions: a nominal pressure of 3.92 MPa, pendulum angle of 90°, and sample mass of 50 mg. As detailed in Table 3, a critical observation emerges: crude NTO samples containing trace nitric acid residues demonstrated significantly elevated sensitivity relative to purified counterparts. Furthermore, non-ultrasonic processed material exhibited broader particle size distribution alongside higher sensitivity metrics. Conversely, the optimized NTO product—featuring reduced particle size and narrow size distribution—manifested a substantial 60 % reduction in impact sensitivity and a concurrent 20 % decrease in friction sensitivity compared to the crude starting material, highlighting the decisive influence of both chemical purity and particulate morphology on insensitivity performance.

**Table 3:** Experimental measurements of the mechanical sensitivity of NTO.

	Impact experiment/%	IS confidence interval	Friction experiment/%	FS confidence interval
Raw	88 %	(69 %,98 %)	44 %	(24 %,65 %)
0 W·cm^−1^	28 %	(12 %,49 %)	16 %	(4 %,36 %)
18 W·cm^−1^	32 %	(15 %,54 %)	16 %	(4 %,36 %)
35 W·cm^−1^	28 %	(12 %,49 %)	20 %	(7 %,41 %)
53 W·cm^−1^	28 %	(12 %,49 %)	20 %	(7 %,41 %)

## 5 Conclusions

This study prepared environmentally friendly NTO crystals exhibiting small particle size, uniform size distribution, and well-defined morphology by controlling the MSZW during the NTO crystallization process. Specifically, the MSZW expands markedly with increasing cooling rate and contracts with elevated solubility. The application of ultrasonic irradiation not only substantially reduces the MSZW but also shows that higher ultrasonic power density further amplifies this effect. Moreover, the ability to form a solid–liquid interface was identified as a key factor governing MSZW variation.

Further analysis based on Sangwal’s nucleation theory—considering parameters such as solid–liquid interfacial tension, critical nucleus size, and crystallization driving force—reveals that ultrasound enhances the driving force for crystallization while concurrently lowering interfacial energy size. These changes facilitate explosiveation and promote the formation of small‐particle‐size NTO crystals. In addition, the morphology of NTO crystals obtained after ultrasonic treatment transforms from rod‐ and a narrower particle size distribution at higher ultrasonic powers. Notably, the ultrasound‐treated NTO exhibits a 60 % reduction in impact sensitivity compared with the raw material, demonstrating superior safety performance.

Although this study elucidates the efficacy of ultrasonic irradiation in the NTO crystallization process and clarifies the mechanisms by which multiple parameters influence crystallization behavior, the ultimate crystal size interplay of nucleation and growth. The pronounced effect of ultrasound on nucleation indicates that, under ultrasonic conditions, crystal size variation is predominantly governed by nucleation, with growth playing a relatively minor role. Future research will focus on assessing the potential hot–spot risks posed by cavitation effects, optimizing ultrasonic irradiation operating parameters under safe conditions, and advancing pilot–scale scale–up, to achieve sustainable and efficient NTO production, thereby providing new insights and directions for the development of crystallization technologies in the field of energetic materials.

Declaration of AI assistance in writing

During the preparation of this manuscript, the authors employed artificial intelligence tools, including gpt-5-mini, to assist with writing, sentence restructuring, and language refinement. These tools were used solely for textual editing purposes, such as improving grammar, punctuation, clarity, and tone. They did not influence the conceptualization of the work or the conduct of the experiments. All authors have thoroughly reviewed and approved the final version of the manuscript to ensure its accuracy and integrity.

## CRediT authorship contribution statement

**Xingquan Hu:** Writing – original draft, Data curation, Conceptualization. **Hao Wu:** Methodology, Funding acquisition, Data curation. **Pei Chang:** Resources, Formal analysis. **Yiying Zhang:** Validation, Resources. **Cheng Xu:** Supervision. **Lianjie Zhai:** Writing – review & editing, Validation, Resources, Project administration. **Bozhou Wang:** Writing – review & editing.

## Declaration of competing interest

The authors declare that they have no known competing financial interests or personal relationships that could have appeared to influence the work reported in this paper. Furthermore, this article does not involve any state secrets.
