# Supplementary material for: The role of ultrasound in the nucleation kinetics and Modification of product properties of 3-Nitro-1,2,4-triazol-5-one
Source: Ultrason Sonochem. 2026 Jan 18;125:107744. doi: 10.1016/j.ultsonch.2026.107744 (PMC12861280; doi:10.1016/j.ultsonch.2026.107744)
Supplement: Supplementary Data 1 [file mmc1.docx]

**Supplementary materials**

**1.1 Computational procedure for determining the molecular volume of NTO.**

$$V_{S}=\frac{M}{\rho N_{A}}\times{10}^{-6} (1)$$

where *M* is the molar mass of the crystal, 130.08 g·mol^-1^; *ρ* is the crystal density, 1.9270 g·cm^-3^; *N*_A_ is the Avogadro constant, 6.02$\times$10^23^mol^-1^. The calculation yields *V_S_* = 1.1213$\times$10^-28^ m^3^.

**1.2 Calculation process of NTO solubilization enthalpy.**

The most common way to calculate dissolution enthalpy is using the Van't Hoff equation:

$$\text{ln}\text{x}\text{=-}\frac{\text{∆}\text{H}_{\text{s}}}{\text{R}_{\text{g}}}\text{∙}\frac{\text{1}}{\text{T}}\text{+C} (2)$$

where *x* is the molar solubility, mol·mol-1; *R_g_* is the gas constant, 8.314 J·mol^-1^·K^-1^. Since the solubility of NTO is nonlinearized with temperature, in order to make the ∆*H_s_* value more consistent with the experimental process, a two-point calculation method is used. Equation 2 transforms to:

$$\Delta H_{S}=-R\cdot\frac{ln\left( \frac{x_{2}}{x_{1}} \right)}{\left( \frac{1}{T_{2}}-\frac{1}{T_{1}} \right)} \left( 3 \right)$$

Substituting the solubility data corresponding to 308.15~328.15 K (reference [8] in the manuscript) yields an average value of Δ*H_s_* of 26.864 kJ·mol^−1^, with a standard deviation of 1.203 kJ·mol^−1^ and a 95% confidence interval of (24.948, 28.779) kJ·mol^−1^.

**1.3 Criteria for judging NTO nucleation**

As shown in Figure S1, cooling was initiated from an initial temperature of 45 °C at a rate of 60 K·h−1; turbidity varied during the nucleation and crystal growth processes. Because at time point B the turbidity curve begins a sustained increase (with an enlarged slope), and white crystals are observed in images captured by the EasyViewer, we define this moment as the nucleation instant (turbidity = 121 NTU).


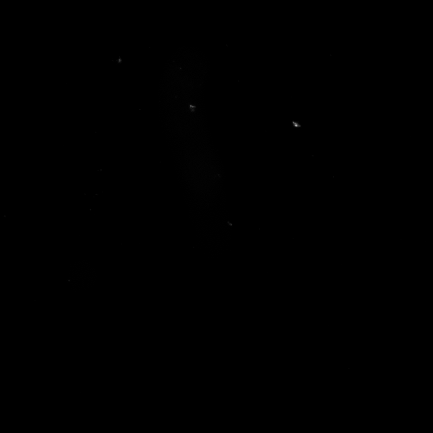

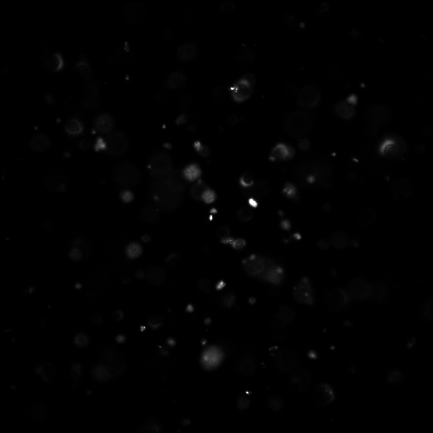

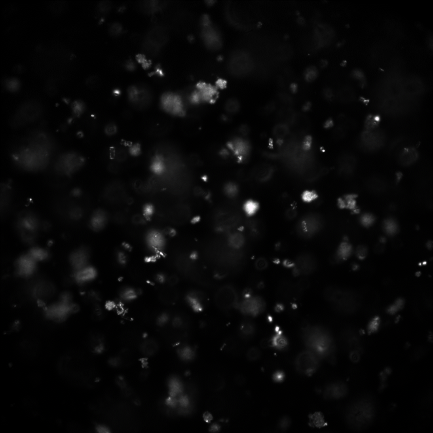

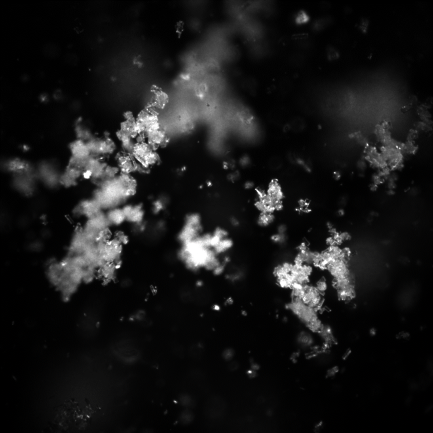


a

b

c

d

**Fig. S1** Schematic diagram for determining the moment of nucleation using a turbidimeter.

**1.4 MSZW raw data**

**Table S1** Raw data of the metastable zone width (MSZW) under non-ultrasound conditions and under ultrasound at 18 W·cm^-1^

|  |  | 0 W·cm^-1^ (*∆T*_max_ /K) | | | | | 18 W·cm^-1^ (*∆T*_max_ /K) | | | | |
| --- | --- | --- | --- | --- | --- | --- | --- | --- | --- | --- | --- |
| R/K·h^-1^ | exp.\T_0_ | 308.15K | 313.15K | 318.15K | 323.15K | 328.15K | 308.15K | 313.15K | 318.15K | 323.15K | 328.15K |
| 6 | 1st | 14.7 | 13.6 | 11.6 | 11.4 | 9.5 | 11.7 | 10.8 | 8.3 | 7.4 | 7.3 |
|  | 2nd | 14.8 | 13.7 | 11.7 | 11 | 9.8 | 11.7 | 10.8 | 8.3 | 7.4 | 7.4 |
|  | 3rd | 14.7 | 13.8 | 11.6 | 11 | 9.7 | 11.7 | 10.8 | 8.3 | 7.5 | 7.3 |
| 30 | 1st | 17.6 | 16.1 | 14.9 | 13.8 | 12.2 | 13.6 | 12.9 | 9.8 | 8.9 | 8.8 |
|  | 2nd | 17.5 | 16.2 | 15.2 | 14.2 | 12 | 13.5 | 12.9 | 9.8 | 8.9 | 8.8 |
|  | 3rd | 17.8 | 16.5 | 15 | 14 | 12 | 13.7 | 12.9 | 9.9 | 9 | 8.8 |
| 60 | 1st | 19.0 | 18.5 | 17 | 16.2 | 14.6 | 14.5 | 14.3 | 10.8 | 9.6 | 9.6 |
|  | 2nd | 19.0 | 18.8 | 17 | 16.3 | 14.4 | 14.6 | 14.2 | 10.9 | 9.7 | 9.5 |
|  | 3rd | 19.0 | 18.7 | 18 | 15.5 | 14.8 | 14.6 | 14.1 | 10.8 | 9.7 | 9.6 |
| 90 | 1st | 21.7 | 20.4 | 19.6 | 17.5 | 15.8 | 16 | 14.9 | 11.8 | 10.8 | 10.5 |
|  | 2nd | 21.8 | 20.4 | 19.6 | 18 | 15.5 | 16 | 14.8 | 11.8 | 10.9 | 10.5 |
|  | 3rd | 21.6 | 20.3 | 19.7 | 17.6 | 16.5 | 16 | 14.9 | 11.8 | 10.9 | 10.5 |

**Table S2** Raw data of the MSZW under ultrasonic conditions of 27 W·cm^-1^ to 80 W·cm^-1^

|  |  | 27 W·cm^-1^ | | | 35 W·cm^-1^ | | | 44 W·cm^-1^ | 53 W·cm^-1^ | 62 W·cm^-1^ | 71 W·cm^-1^ | 80 W·cm^-1^ |
| --- | --- | --- | --- | --- | --- | --- | --- | --- | --- | --- | --- | --- |
| R/K·h^-1^ | exp.\T0 | 308.15K | 313.15K | 318.15K | 308.15K | 313.15K | 318.15K | 318.15K | 318.15K | 318.15K | 318.15K | 318.15K |
| 6 | 1st | 11.7 | 10.8 | 8.3 | 11.7 | 10.8 | 8.3 | 8.1 | 7.2 | 7.3 | 6.7 | 6.7 |
|  | 2nd | 11.6 | 10.8 | 8.2 | 11.6 | 10.8 | 8.2 | 8.2 | 7.2 | 7.3 | 6.8 | 6.8 |
|  | 3rd | 11.7 | 10.7 | 8.2 | 11.7 | 10.7 | 8.3 | 8.1 | 7.3 | 7.1 | 6.8 | 6.7 |
| 30 | 1st | 13.6 | 12.9 | 9.8 | 13.6 | 13 | 9.8 | 9.8 | 8.6 | 8.5 | 7.5 | 7.7 |
|  | 2nd | 13.5 | 12.9 | 9.8 | 13.5 | 12.9 | 9.8 | 9.7 | 8.6 | 8.4 | 7.7 | 7.6 |
|  | 3rd | 13.7 | 12.9 | 9.9 | 13.7 | 12.9 | 9.9 | 9.8 | 8.6 | 8.6 | 7.6 | 7.4 |
| 60 | 1st | 14.5 | 14.3 | 10.8 | 14.5 | 14.3 | 10.8 | 10.7 | 9.4 | 9.2 | 8.5 | 8.5 |
|  | 2nd | 14.6 | 14.2 | 10.9 | 14.6 | 14.1 | 10.9 | 10.8 | 9.4 | 9.4 | 8.6 | 8.7 |
|  | 3rd | 14.6 | 14.1 | 10.8 | 14.6 | 14.1 | 10.8 | 10.7 | 9.4 | 9.5 | 8.4 | 8.4 |
| 90 | 1st | 16 | 15 | 11.8 | 16 | 15 | 11.8 | 11.5 | 10.5 | 10.5 | 9.6 | 9.7 |
|  | 2nd | 16.1 | 14.8 | 11.8 | 16.1 | 14.8 | 11.6 | 11.7 | 10.4 | 10.4 | 9.7 | 9.6 |
|  | 3rd | 16.1 | 14.9 | 11.7 | 16.1 | 14.9 | 11.8 | 11.8 | 10.5 | 10.3 | 9.7 | 9.7 |

**1.5 Analysis of the dispersion characteristics of MSZW raw data**

**Table S3** Uncertainty and confidence intervals of MSZW data obtained under non-ultrasonic and under 18 W·cm^-1^ ultrasonic conditions

|  | (*∆T*_max_/K) | 0 W·cm-1 (∆Tmax /K) | | | | | 18 W·cm-1 (∆Tmax /K) | | | | |
| --- | --- | --- | --- | --- | --- | --- | --- | --- | --- | --- | --- |
| R/K·h-1 | Analysis | 308.15K | 313.15K | 318.15K | 323.15K | 328.15K | 308.15K | 313.15K | 318.15K | 323.15K | 328.15K |
| 6 | U^[[1]](#footnote-1)^ | 0.1434 | 0.2484 | 0.1434 | 0.5737 | 0.3795 | 0.0000 | 0.0000 | 0.0000 | 0.1434 | 0.1434 |
|  | UCL^[[2]](#footnote-2)^ | 14.88 | 13.95 | 15.41 | 11.71 | 10.05 | 11.70 | 10.80 | 8.30 | 7.58 | 7.48 |
|  | LCL^[[3]](#footnote-3)^ | 14.59 | 13.45 | 14.65 | 10.56 | 9.29 | 11.70 | 10.80 | 8.30 | 7.29 | 7.19 |
| 30 | U | 0.3795 | 0.5171 | 0.3795 | 0.4968 | 0.2868 | 0.2484 | 0.0000 | 0.1434 | 0.1434 | 0.0000 |
|  | UCL | 18.01 | 16.78 | 15.20 | 14.50 | 12.35 | 13.85 | 12.90 | 9.98 | 9.08 | 8.80 |
|  | LCL | 17.25 | 15.75 | 15.00 | 13.50 | 11.78 | 13.35 | 12.90 | 9.69 | 8.79 | 8.80 |
| 60 | U | 0.0000 | 0.3795 | 1.4342 | 1.0828 | 0.4968 | 0.1434 | 0.2484 | 0.1434 | 0.1434 | 0.1434 |
|  | UCL | 19.00 | 19.05 | 18.77 | 17.08 | 15.10 | 14.71 | 14.45 | 10.98 | 9.81 | 9.71 |
|  | LCL | 19.00 | 18.29 | 15.90 | 14.92 | 14.10 | 14.42 | 13.95 | 10.69 | 9.52 | 9.42 |
| 90 | U | 0.2484 | 0.1434 | 0.1434 | 0.6572 | 1.2748 | 0.0000 | 0.1434 | 0.0000 | 0.1434 | 0.0000 |
|  | UCL | 21.95 | 20.51 | 19.78 | 18.36 | 17.21 | 16.00 | 15.01 | 11.80 | 11.01 | 10.50 |
|  | LCL | 21.45 | 20.22 | 19.49 | 17.04 | 14.66 | 16.00 | 14.72 | 11.80 | 10.72 | 10.50 |

**Table S4** Uncertainty and confidence intervals under ultrasonic conditions in the range of 27 W·cm^-1^ to 80 W·cm^-1^.

|  | (*∆T*_max_ /K) | 27 W·cm^-1^ | | | 35 W·cm^-1^ | | | 44 W·cm^-1^ | 53 W·cm^-1^ | 62 W·cm^-1^ | 71 W·cm^-1^ | 80 W·cm^-1^ |
| --- | --- | --- | --- | --- | --- | --- | --- | --- | --- | --- | --- | --- |
| R/K·h^-1^ | Analysis | 308.15K | 313.15K | 318.15K | 308.15K | 313.15K | 318.15K | 318.15K | 318.15K | 318.15K | 318.15K | 318.15K |
| 6 | U | 0.1434 | 0.1434 | 0.1434 | 0.1434 | 0.1434 | 0.1434 | 0.1434 | 0.1434 | 0.2868 | 0.1434 | 0.1434 |
|  | UCL | 11.81 | 10.91 | 8.38 | 11.81 | 10.91 | 8.41 | 8.28 | 7.38 | 7.52 | 6.91 | 6.88 |
|  | LCL | 11.52 | 10.62 | 8.09 | 11.52 | 10.62 | 8.12 | 7.99 | 7.09 | 6.95 | 6.62 | 6.59 |
| 30 | U | 0.2484 | 0.0000 | 0.1434 | 0.2484 | 0.1434 | 0.1434 | 0.1434 | 0.0000 | 0.2484 | 0.2484 | 0.3795 |
|  | UCL | 13.85 | 12.90 | 9.98 | 13.85 | 13.08 | 9.98 | 9.91 | 8.60 | 8.75 | 7.85 | 7.95 |
|  | LCL | 13.35 | 12.90 | 9.69 | 13.35 | 12.79 | 9.69 | 9.62 | 8.60 | 8.25 | 7.35 | 7.19 |
| 60 | U | 0.1434 | 0.2484 | 0.1434 | 0.1434 | 0.2868 | 0.1434 | 0.1434 | 0.0000 | 0.3795 | 0.2484 | 0.3795 |
|  | UCL | 14.71 | 14.45 | 10.98 | 14.71 | 14.45 | 10.98 | 10.88 | 9.40 | 9.75 | 8.75 | 8.91 |
|  | LCL | 14.42 | 13.95 | 10.69 | 14.42 | 13.88 | 10.69 | 10.59 | 9.40 | 8.99 | 8.25 | 8.15 |
| 90 | U | 0.1434 | 0.2484 | 0.1434 | 0.1434 | 0.2484 | 0.2868 | 0.3795 | 0.1434 | 0.2484 | 0.1434 | 0.1434 |
|  | UCL | 16.21 | 15.15 | 11.91 | 16.21 | 15.15 | 12.02 | 12.05 | 10.61 | 10.65 | 9.81 | 9.81 |
|  | LCL | 15.92 | 14.65 | 11.62 | 15.92 | 14.65 | 11.45 | 11.29 | 10.32 | 10.15 | 9.52 | 9.52 |

**Table S5** Statistical significance testing of differences in MSZW data between the non-ultrasound condition

and the ultrasonic condition at 18 W·cm^-1^

|  | 308.15K | 313.15K | 318.15K | 323.15K | 328.15K |
| --- | --- | --- | --- | --- | --- |
| P | 0.0001 | 0.0002 | 0.00003 | 0.000006 | 0.0001 |
| Sig. diff. | ＜0.001 | ＜0.001 | ＜0.001 | ＜0.001 | ＜0.001 |

Compare the MSZW measurements between non‑ultrasonic conditions and ultrasonic conditions at 18 W·cm^-1, and evaluate the statistical significance of their differences. At *T*_0_ = 308.15 K, the p value was 0.0001 (< 0.001); the MSZW measurements under ultrasonic conditions at 18 W·cm^-1^ were significantly lower than those under non‑ultrasonic conditions, and the difference was highly statistically significant. Similar results were obtained at other temperatures.

1. U：Uncertainty [↑](#footnote-ref-1)
2. UCL: Upper confidence limit [↑](#footnote-ref-2)
3. LCL: Lower confidence limit [↑](#footnote-ref-3)
